# Bloody Diarrhea Associated with Hookworm Infection in Traveler Returning to France from Myanmar

**DOI:** 10.3201/eid2110.150695

**Published:** 2015-10

**Authors:** Julie Brunet, Jean-Philippe Lemoine, Nicolas Lefebvre, Julie Denis, Alexander W. Pfaff, Ahmed Abou-Bacar, Rebecca J. Traub, Bernard Pesson, Ermanno Candolfi

**Affiliations:** Hôpitaux Universitaires de Strasbourg, Strasbourg, France (J. Brunet, J.-P. Lemoine, N. Lefebvre, J. Denis, A.W. Pfaff, A. Abou-Bacar, B. Pesson, E. Candolfi);; Université de Strasbourg, Strasbourg (J. Brunet, A.W. Pfaff, E. Candolfi);; University of Melbourne, Parkville, Victoria, Australia (R.J. Traub)

**Keywords:** Ancylostoma ceylanicum, hookworm, infection, bloody diarrhea, tourist, parasites, zoonoses, Myanmar

**To the Editor:** Human hookworm infections are commonly caused by 2 anthroponotic species, *Necator americanus* and *Ancylostoma duodenale*. However, *A. ceylanicum*, a zoonotic hookworm of canids and felids, is emerging as the second most common human hookworm in Southeast Asia (*1**–**4*). Two haplotypes of *A*. *ceylanicum* hookworm have been identified, 1 specific to humans and 1 specific to humans, dogs, and cats (*4**,**5*). We report a case of patent enteric *A. ceylanicum* hookworm infection in a man from France who had visited Myanmar.

In December 2014, a 33-year-old man with no medical history sought care in France after 3 weeks of fever, vomiting, dyspnea, bloody diarrhea, and weight loss (7 kg). He had returned from a 3-week trip to Myanmar 1 month earlier. Two days after his arrival in Myanmar, he had pruritic erythematous macules on the buttocks after sitting in a public park in Rangoon while wearing short pants; this sign was followed by a dry cough.

Laboratory data showed leukocytosis (17.43 × 10^9^ cells/L) with hypereosinophilia (55%) and a hemocrit of 56.1%. Direct examination of hemorrhagic stool showed numerous Charcot-Leyden crystals (CLCs) and 150–200 eggs/g feces of unembryonated hookworm ova (mean size 57.6 × 38.4 µm) (Figure, panel A). Rhabditiform and filariform larvae were isolated by stool culture (Figure, panel B). On the basis of clinical history and data suggestive of eosinophilic enteritis, which is uncommon in patients infected with parasites adapted to humans, a zoonotic hookworm species was suspected.

**Figure:**
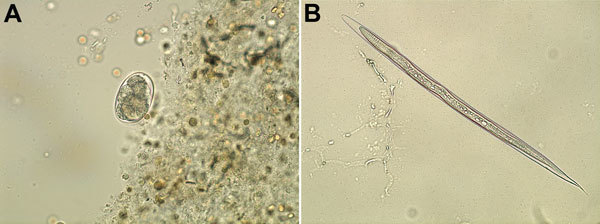
*Ancylostoma ceylanicum* hookworm isolated from a French tourist returning from Myanmar. A) Ova and blood cells in fecal specimen. B) Filiform larvae after stool culture. Original magnifications ×40.

For species identification, DNA was extracted from larvae by using the DNeasy Tissue Kit (QIAGEN, Hilden, Germany) at the Faculty of Veterinary and Agricultural Sciences, University of Melbourne (Parkville, Victoria, Australia) and subjected to PCR specific for the ribosomal internal transcribed spacer region of hookworms (*1*). Testing was conducted at the University of Melbourne because this institution has the technical expertise for identifying hookworm. In addition, haplotype characterization was performed by using PCR specific for the mitochondrial cytochrome oxidase-1 gene (*4*). Bidirectional DNA sequences of PCR products (Macrogen Inc., Seoul, South Korea) were analyzed by using Finch TV 1.4.0 (Geospiza Inc., Seattle, WA, USA). The ribosomal internal transcribed spacer region had 100% sequence identity with an *A. ceylanicum* sequence in GenBank (DQ381541). Neighbor-joining analyses with MEGA 4.1 (http://www.megasoftware.net) clustered the isolate within the *A. ceylanicum* haplotype specific for animals and humans (*4*).

By the third day of albendazole therapy (400 mg/d), clinical improvement was observed and stool specimens were negative for hookworm ova. However, eosinophilia (59%) was persistent. Three months later, the patient was hospitalized because of diarrhea and abdominal pain. CLCs and hookworm ova were observed, and the patient received a second course of albendazole.

*A. ceylanicum* hookworm was unequivocally identified by using molecular methods as the etiologic cause of the original signs and symptoms for this patient. In Asia, *A. ceylanicum* hookworm is reported at a prevalence of 62%–92% in stray dogs and cats (*3*) and environmental contamination of the public park by dog feces was the probable source of infection for this patient.

*A. ceylanicum* hookworms have been experimentally shown to develop to patency in humans within 26–35 days (*6*), but abdominal symptoms and eosinophilia may occur earlier (within 21 days). Natural human infections have been described in most regions where *A. ceylanicum* hookworm is endemic to animals, but clinical and pathologic data are scarce. In Taiwan and Malaysia, this hookworm was visualized in the mid-jejunum in patients with acute, severe abdominal pain and nausea and in terminal ileum in patients with anemia caused by chronic blood loss, nausea, and melena (*7**–**9*).

In all cases, leukocytosis with eosinophilia (22%–50%) was observed. In the patient we describe, transdermal infection causing cutaneous larva migrans was followed by development of eosinophilic enteritis within a 2-week period. Enteric signs were similar to those observed in previous cases in Taiwan. A 3-day course of benzimidazole is the anthelminthic drug of choice. However, clinicians must be aware of possible relapse, potentially caused by failure of the adulticidal drug to kill developing larvae before full patency is reached (*3**,**8**,**9*).

Differentiation of larvae and eggs of anthroponotic and zoonotic *Ancylostoma* spp. is difficult. Definitive diagnosis relies on detailed morphologic identification of adult worms or molecular identification of adults, eggs, or larvae in stool specimens. Thus, human infection in travelers returning from parasite-endemic regions is likely to be misdiagnosed as an anthroponotic hookworm species. Clinically, the presence of hookworm ova and an unusually high number of CLCs and eosinophils in stool should alert clinicians to the possibility of an infection with *A. ceylanicum* hookworm.

*A. ceylanicum* hookworm is reported primarily in tropical climates; the possibility of spread in temperate countries remains low because development of filariforme larva requires high temperatures and a moist environment. However, recent climate changes, coupled with poor sanitary conditions, could promote emergence of tropical species, and recently, rare cases of autochthonous hookworm-related cutaneous larva migrans have been reported in Europe (*10*).

This report highlights the risk for zoonotic ancylostomiasis in travelers visiting countries to which *A. ceylanicum* hookworm is endemic among animals. It also emphasizes the usefulness of copromolecular techniques for species-specific diagnosis.
